# Computed tomography characterization and outcome evaluation of COVID-19 pneumonia complicated by venous thromboembolism

**DOI:** 10.1371/journal.pone.0242475

**Published:** 2020-11-19

**Authors:** Stefanie Meiler, Okka Wilkea Hamer, Jan Schaible, Florian Zeman, Niels Zorger, Henning Kleine, Janine Rennert, Christian Stroszczynski, Florian Poschenrieder

**Affiliations:** 1 Department of Radiology, Regensburg University Medical Center, Regensburg, Germany; 2 Department of Radiology, Hospital Donaustauf, Donaustauf, Germany; 3 Center for Clinical Studies, Regensburg University Medical Center, Regensburg, Germany; 4 Department of Radiology, Hospital Barmherzige Brueder, Regensburg, Germany; 5 Department of Pneumology, Hospital Barmherzige Brueder, Regensburg, Germany; University of Western Ontario, CANADA

## Abstract

**Background:**

COVID-19 is frequently complicated by venous thromboembolism (VTE). Computed tomography (CT) of the chest—primarily usually conducted as low-dose, non-contrast enhanced CT—plays an important role in the diagnosis and follow-up of COVID-19 pneumonia. Performed as contrast-enhanced CT pulmonary angiography, it can reliably detect or rule-out pulmonary embolism (PE). Several imaging characteristics of COVID-19 pneumonia have been described for chest CT, but no study evaluated CT findings in the context of VTE/PE.

**Purpose:**

In our retrospective study, we analyzed clinical, laboratory and CT imaging characteristics of 50 consecutive patients with RT-PCR proven COVID-19 pneumonia who underwent contrast-enhanced chest CT at two tertiary care medical centers.

**Material and methods:**

All patients with RT-PCR proven COVID-19 pneumonia and contrast-enhanced chest CT performed at two tertiary care hospitals between March 1st and April 20th 2020 were retrospectively identified. Patient characteristics (age, gender, comorbidities), symptoms, date of symptom onset, RT-PCR results, imaging results of CT and leg ultrasound, laboratory findings (C-reactive protein, differential blood count, troponine, N-terminal pro-B-type natriuretic peptide (NT-proBNP), fibrinogen, interleukin-6, D-dimer, lactate dehydrogenase (LDH), creatine kinase (CK), creatine kinase muscle-brain (CKmb) and lactate,) and patient outcome (positive: discharge or treatment on normal ward; negative: treatment on intensive care unit (ICU), need for mechanical ventilation, extracorporeal membrane oxygenation (ECMO), or death) were analyzed. Follow-up was performed until May 10th. Patients were assigned to two groups according to two endpoints: venous thromboembolism (VTE) or no VTE. For statistical analysis, univariate logistic regression models were calculated.

**Results:**

This study includes 50 patients. In 14 out of 50 patients (28%), pulmonary embolism was detected at contrast-enhanced chest CT. The majority of PE was detected on CTs performed on day 11–20 after symptom onset. Two patients (14%) with PE simultaneously had evidence of deep vein thrombosis. 15 patients (30%) had a negative outcome (need for intensive care, mechanical ventilation, extracorporeal membrane oxygenation, or death), and 35 patients (70%) had a positive outcome (transfer to regular ward, or discharge). Patients suffering VTE had a statistically significant higher risk of an unfavorable outcome (p = 0.028). In univariate analysis, two imaging characteristics on chest CT were associated with VTE: crazy paving pattern (p = 0.024) and air bronchogram (n = 0.021). Also, elevated levels of NT-pro BNP (p = 0.043), CK (p = 0.023) and D-dimers (p = 0.035) were significantly correlated with VTE.

**Conclusion:**

COVID-19 pneumonia is frequently complicated by pulmonary embolism (incidence of 28% in our cohort), remarkably with lacking evidence of deep vein thrombosis in nearly all thus affected patients of our cohort. As patients suffering VTE had an adverse outcome, we call for a high level of alertness for PE and advocate a lower threshold for contrast-enhanced CT in COVID-19 pneumonia. According to our observations, this might be particularly justified in the second week of disease and if a crazy paving pattern and / or air bronchogram is present on previous non-enhanced CT.

## Introduction

SARS-CoV-2-infection (Severe Acute Respiratory Syndrome Coronavirus 2) / COVID-19 (Coronavirus Disease 2019) is associated with a wide spectrum of clinical presentation, ranging from mild symptoms in the majority of cases [1–4] to severe and even fatal disease courses in approximately 10% of patients [5]. Severe courses of COVID-19 are often complicated by acute respiratory distress syndrome and multi-organ failure. Moreover, the patients are prone to thromboembolic complications with venous thromboembolism (VTE) being the commonest. The reported frequencies of pulmonary embolism (PE) detected by contrast-enhanced computed tomography (CT) range from 23 to 40% [6–10]. However, international guidelines recommend non-enhanced low dose CT for evaluation of COVID-19 pneumonia [11, 12]. Hence, thromboembolic complications might be missed and mandatory therapy withheld.

The aim of our retrospective study was to evaluate parenchymal characteristics in chest CT of COVID-19 pneumonia and laboratory parameters which are associated with pulmonary embolism or venous thrombosis. Furthermore, we analyzed the clinical outcome of patients with COVID-19 pneumonia with and without VTE.

## Material and methods

### Patient population

The study was approved by the institutional Ethics Committee of the University Medical Center Regensburg (IRB No. 20-1784-104). Written informed consent was waived. All procedures performed in studies involving human participants were in accordance with the ethical standards of the institutional and / or national research committee and with the 1964 Helsinki declaration and its later amendments or comparable ethical standards.

The inclusion criteria were consecutive adult patients (≥ 18 years old) with RT-PCR (real transcription polymerase chain reaction) positive for SARS-CoV-2 and a contrast-enhanced chest CT performed between March 1st and April 20th 2020. Exclusion criteria were a negative result of RT-PCR for SARS-CoV-2 and non-diagnostic CT for example due to motion-artifacts. Patients were identified by means of a full-text database query of all CT-scans performed between March 1st and April 20th 2020 using the term “*COVID*” OR *SARS* in the Radiological Information System (RIS, Nexus.medRIS, Version 8.42, Nexus, Villingen-Schwenningen, Germany). Patient characteristics (age, gender, comorbidities), symptoms, date of symptom onset, RT-PCR results, imaging results of leg ultrasound (if performed), laboratory findings (C-reactive protein, differential blood count, troponine, N-terminal pro-B-type natriuretic peptide(NT-proBNP), fibrinogen, interleukin-6, D-dimer, LDH, CK, CKmb and lactate) and patient outcome were extracted from electronic patient records. As for laboratory parameters the results determined closest to the date of the chest CT scan but not more than 3 days before or after were evaluated. All patients had at least one contrast-enhanced CT scan of the chest. Out of these CT scans, we included the first scan per patient. Abdominal CT, if performed, was included if scan date was not more than 3 days before or after the chest CT. Patients were followed until May 10th 2020.

### CT protocol

The patients underwent CT scans at two tertiary care hospitals and CTs were performed on two different scanners (16 slice Somatom Sensation 16, 128-slice Definition FLASH, Siemens Healthcare, Forchheim, Germany). All chest CT acquisitions were obtained in supine position during end-inspiration. The contrast media used was Accupaque 300 or 350 in a weight adapted dose for chest CT (but not more than 70) and 100 ml preset for abdomen CT intravenously administered at a flow rate of 4 ml/s (chest) or 3 ml/s (abdomen). Automatic tube voltage selection was applied with a reference tube voltage of 80–140 kV (CARE kV, Siemens Healthcare, Erlangen, Germany). Tube current was regulated by an automatic tube current modulation technique with the reference mAs setting being 130-150mAs (chest) or 210 mAs (abdomen). Collimation width was 0.625 mm-0.75 mm. Multiplanar reformations (MPR) were reconstructed in the transversal plane with a slice thickness of 0.75–1.5 mm (48 CTs) and 3 mm (2 CTs) in lung kernel and with a slice thickness of 0,75 mm (n = 3), 1 mm (n = 41), 3 mm (n = 3) and 5 mm (n = 3)in soft tissue kernel. Additional sagittal and coronal MPR were reconstructed with a slice thickness of 1–3 mm using lung and soft tissue kernel. The images were sent to a picture archiving and communication system (PACS, Syngo Imaging, Siemens Healthcare, Erlangen, Germany).

### Image analysis

Two junior radiologists with subspeciality training in thoracic radiology evaluated each half of the CT studies on a PACS workstation. The evaluated patterns had been part of the training of the radiologists. In equivocal cases a senior thoracic radiologist was consulted.

The radiologists were blinded to clinical data, laboratory data and patient status. The Fleischner Society definition of CT features were applied when appropriate. The following parameters were analysed:

- ground-glass-opacities (GGO): hazy increased opacity of lung, with preservation of bronchial and vascular margins.
- consolidation: homogeneous increase in pulmonary parenchymal attenuation that obscures the margins of vessels and airway walls
- crazy-paving pattern: thickened interlobular septa and intralobular lines superimposed on a background of ground-glass opacity.
- cavity: gas-filled space within consolidation
- bronchial dilatation: dilated (with respect to the accompanying pulmonary artery) non-tapering bronchus. The term „dilatation”instead of „-ectasis”was intentionally used in order to express that the pathology might be reversible.
- vessel dilatation: diameter of vessel within or near opacifications clearly larger compared to vessels of the same generation in healthy lung tissue.
- shape of opacification: round, curvilinear/band-like, geographic (= opacification outlines the shape of multiple adjacent pulmonary lobules, sharply marginated)
- margin of opacification: unsharp, at least to some extent sharp
- lung lobes affected
- distribution of opacifications in the axial plane: predominantly peripheral, predominantly central, predominantly anterior, predominantly posterior, diffuse
- lymphadenopathy: diameter > 10 mm in short axis.
- subjective estimation of extent of parenchymal opacification: 0–33%: mild, 34–66%: moderate, 67–100%: severe.
- pulmonary embolism (PE): contrast medium filling defects within the pulmonary arteries
- deep vein thrombosis (DVT) in CT: contrast medium filling defect in iliacal and / or femoral veins
- DVT in US: incomplete compressibility and absence of blood flow in iliacal veins and / or veins of the lower extremity

### Definition of outcome

Positive outcome was defined as either discharge or regular ward care, and negative outcome was defined to be need for intubation / mechanical ventilation and / or need for treatment on intensive care unit and / or need for extracorporeal membrane oxygenation (ECMO), or death.

### Statistical analysis

Age is presented as mean (standard deviation) while all laboratory variables and time to onset are presented as median (interquartile range) due to their skewed distribution. All categorical variables are presented as absolute and relative frequencies. Differences between patients with and without a venous thromboembolism (primary endpoint) were analyzed for all parameters (demographic data, clinical data, comorbidities and CT findings) by using the Mann-Whitney-U Test for all continuous variables and the Chi-square test of independence for all categorical variables. A multivariable model was not feasible due to the limited number of patients and events. A p-value <0.05 was considered statistically significant. All analyses were performed using R, version 3.6.1 (The R Foundation for Statistical Computing).

## Results

### Patient population and clinical symptoms

The study population consisted of 50 patients (male: 34(68%)) with a mean age of 60.4 years (10.1SD, range 37–88). A total of 59contrast-enhanced CT scans were performed. The first CT performed per patient was included into the analysis. Thus, 50 CTs were evaluated, including 24 sole chest scans and 26 combined chest/abdomen scans. A total of 4 ultrasound examinations of the lower extremities were performed due to clinical suspicion for deep venous thrombosis. Two of these were patients with sole chest scans, the other 2 were patients with combined chest / abdomen scans.

Clinical symptoms were fever in 37 patients (74%), cough in 34 patients (68%), dyspnea in 34 patients (68%) and fatigue in 23 patients (46%). Less frequently were gastrointestinal complaints (n = 13; 26%) and taste dysfunction (n = 8; 16%). Most common comorbidities were high blood pressure (n = 20, 40%) and diabetes (n = 10, 20%). Prevalence of obesity and smoking history could not be determined because of under-report of corresponding data in patient charts.

15 patients (30%) had a positive outcome, and 35 patients (70%) had a negative outcome.

**Fig 1:**
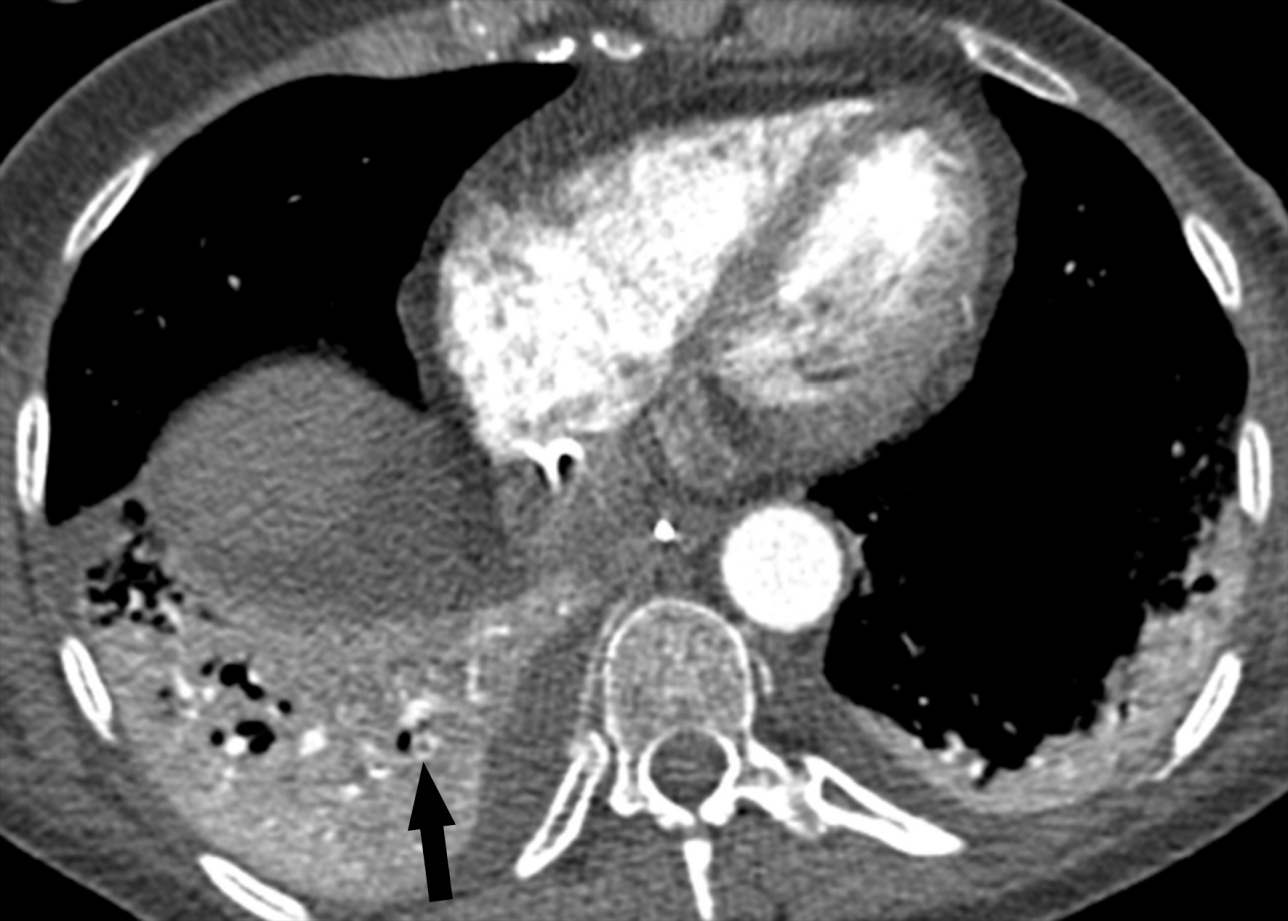
Pulmonary embolism. Axial reconstruction of a CT in soft tissue window showing pulmonary embolism of the right lower lobe.

14 of 50 patients (28%) had evidence of PE as detected by chest CT (Fig 1). Two of these patients also had deep venous thrombosis confirmed in one case by abdominal CT and in the other by ultrasound. These patients were assigned to group 1. The remainder (36/50 patients, 72%) without VTE was assigned to group 2. No patient had venous thrombosis but no PE. The results are presented in Table 1.

**Table 1:** Patient characteristics (n = 50).

patient characteristics	patients (n = 50)
**age (years)**	60.4 (SD 10.1)
**gender**	
male	34 (68%)
female	16 (34%)
**symptoms**	
fever* (>37.5°C)	37 (74%)
cough*	34 (68%)
dyspnea*	34 (68%)
fatigue*	23(46%)
taste dysfunction*	8 (16%)
gastrointestinal*	13 (26%)
**Comorbidities**	
diabetes*	10 (20%)
cardiac failure*	2 (4%)
coronary heart disease*	4 (8%)
COPD*	2 (4%)
asthma*	3 (6%)
high blood pressure*	20 (40%)
**outcome**	
positive outcome	15 (30%)
negative outcome	25 (70%)
**incidence of VTE**	
VTE (group 1)	14 (28%)
no VTE (group 2)	36 (72%)
**time between symptom onset and CT scan (days)**	15 (IQR 8.5–24.5)**
0–10	13 (33%)
11–20	13 (33%)
21–30	7 (18%)
> 30	6 (15%)

* occasional missing values

** for 11 patients symptom onset was not documented

COPD = chronic obstructive pulmonary disease, VTE = venous thromboembolism

### CT morphology

Ground glass opacities (96%, n = 48) and consolidation (86%, n = 43) were the most commonly observed patterns on chest CT. A crazy paving pattern was identified in 46% of the scans (n = 23). Bronchial dilatation was seen in 20% (n = 10), air bronchogram in 60% (n = 30) and vessel enlargement in 56% (n = 28) of CTs. Pleural effusion was observed in 30% (n = 15) and lymphadenopathy in 58% (n = 29) of CTs.

Most of the lesions were to at least some extent sharply marginated (74%, n = 37). In 36% of CTs (n = 18), opacifications were curvilinear shaped or round, and geographic in 24% (n = 12). The vast majority of CTs showed pulmonary lesions bilaterally (94%, n = 47), and pulmonary lobes were affected equally frequent with slight preponderance of lesions in the right lower lobe, left lower lobe and left upper lobe (n = 47, 94% each) as compared to the remaining lobes (n = 45, 90%). Distribution in the axial plane was predominantly posterior (78%, n = 39). Lesions were predominantly peripherally located in 26 patients (52%) and diffusely distributed in 24 patients (48%). A mild extent of opacifications was seen in 17% (n = 8) of CTs, moderate extent in 12% (n = 6), and severe extent in 71% (n = 34). Results are presented in Table 2.

**Table 2:** CT characterization of pulmonary pathology.

CT findings	patients n = 50
**CT signs**	
consolidation	43 (86%)
GGO	48 (96%)
crazy paving	23 (46%)
round shape of opacification	18 (36%)
sharp margin of opacification	37 (74%)
geographic shape of opacification	12 (24%)
curvilinear/bandlike opacification	18 (36%)
bronchial dilatation	20 (10%)
air bronchogram	60 (30%)
cavitation	0 (0%)
peripheral vessel enlargement	28 (56%)
pleural effusion	15 (30%)
lymphadenopathy	29 (58%)
**distribution**	
bilateral	47 (94%)
unilateral	1 (2%)
right upper lobe	45 (90%)
right middle lobe	45 (90%)
right lower lobe	47 (94%)
left upper lobe	47 (94%)
left lower lobe	47 (94%)
predominantly anterior	0 (0%)
predominantly posterior	39 (78%)
predominantly peripheral	26 (52%)
diffuse	24 (48%)
**extent of lung involvement**	
mild	8 (12%)
moderate	6 (12%)
severe	34 (71%)

CT = computed tomography, GGO = ground-glass opacities

### Laboratory parameters

Laboratory analysis revealed an elevated C-reactive protein (CRP)with a median of 127 mg/l (IQR 57.17–235.75), lactate dehydrogenase (LDH) with a median of 392 U/l (IQR 299.5–477.75), interleukin 6 with a median of 149.8 pg/ml (IQR 58.6–511.5), but also troponine (median 30.2 (IQR 14.02–65.58)) and N-terminal pro-B-type natriuretic peptide (NT-proBNP) (median 873.5 (IQR 455.25–2748.75)). Fibrinogen had a median of 581.3 mg/dl (IQR 495.8–660.4), D-dimers a median of 2.92 mg/l (IQR 1.3–6.88). These parameters are presented in Table 3.

**Table 3:** Laboratory parameters.

parameter	n	median	normal range
CRP (mg/l)	48	127 (IQR 57.17–235.75)	< 5
leukocytes (/nl)	48	9.63 (IQR 6.77–13.9)	4.23–9.1
lymphocytes rel (%)	42	12.35 (IQR 7.77–21.1)	21.5–53.1
lymphocytes abs	38	1.08 (IQR 0.8–1.53)	1.32–3.57
eosinophiles rel (%)	40	0.35 (IQR 0–1.1)	0.8–7
eosinophiles abs	38	0.04 (IQR 0–0.12)	0.04–0.54
troponine (ng/l)	34	30.2 (IQR 14.02–65.58)	< = 14
NT-proBNP (pg/ml)	18	873.5 (IQR 455.25–2748.75)	<486
fibrinogen (mg/dl)	32	581.3 (IQR 495.8–660.4)	210–400
interleukin 6 (pg/ml)	32	149.8 (IQR 58.6–511.5)	<7
D-dimer (mg/l)	38	2.92 (IQR 1.3–6.88)	<0.5
LDH (U/I)	44	392 (IQR 299.5–477.75)	<250
CK (U/I)	44	172 (IQR 57.5–373.25)	<190
CKmb (ng/ml)	26	2.85 (IQR 1.72–9.57)	0.5–3.6
lactate (mg/dl)	39	11 (IQR 9–14.5)	<16

CRP = C reactive protein, NT-proBNP = N-terminal pro-B-type natriuretic peptide, LDH = lactate dehydrogenase, CK = creatin-kinase, CKmb = creatin-kinase muscle-brain

### Univariate analysis for identification of parameters with impact on patient’s outcome

#### Demographic data, clinical data and comorbidities

Symptoms of taste dysfunction at first admission were exclusively present in patients without evidence of VTE, resulting in a statistically significant difference in the frequency of this clinical symptom between the two groups with / without VTE (p = 0.02, Table 4). No other demographic or clinical parameters and comorbidities influenced the event of VTE. Gender distribution in both groups was comparable (p = 0.70).

**Table 4:** Differences between VTE vs. no VTE in demographic data, clinical data, comorbidities and outcome.

parameter	VTE group (n = 14)	no VTE group (n = 36)	p-value
age	60.5(SD 9.7)	76.1(SD 94.5)	0.543
gender			
male	10 (30%)	23 (70%)	
female	4 (25%)	12 (75%)	0.700
symptoms			
fever*			
No	4 (50%)	4 (50%)	
Yes	8 (22%)	29 (78%)	0.100
cough*			
no	2 (20%)	8 (80%)	
yes	9 (26%)	25 (74%)	0.678
dyspnoea*			
no	4 (40%)	6 (60%)	
yes	10 (29%)	24 (71%)	0.527
fatigue*			
no	6 (46%)	7 (54%)	
yes	6 (26%)	17 (74%)	0.220
gastrointestinal*			
no	10 (45%)	12 (55%)	
yes	2 (15%)	11 (85%)	0.070
taste dysfunction*			
no	12 (44%)	15 (56%)	
yes	0 (0%)	8 (100%)	**0.020**
comorbidities			
diabetes*			
no	10 (25%)	30 (75%)	
yes	4 (40%)	6 (60%)	0.345
cardiac failure*			
no	14 (29%)	34 (71%)	
yes	0 (0%)	2 (100%)	0.368
coronary heart disease*			
no	14 (30%)	32 (70%)	
yes	0 (0%)	4 (100%)	0.193
COPD*			
no	14 (29%)	34 (71%)	
yes	0 (0%)	2 (100%)	0.368
asthma*			
no	14 (30%)	33 (70%)	
yes	0 (0%)	3 (100%)	0.265
high blood pressure*			
no	9 (30%)	21 (70%)	
yes	5 (25%)	15 (75%)	0.700
outcome			
negative	13 (92.9%)	22 (61.1%)	
positive	1 (7.1%)	14 (38.9%)	**0.028**
**time between symptom onset and CT scan (days)**	16 (IQR 13.5–20)	15 (IQR 7–25)	0.326**
0–10	1 (8%)	12 (92%)	reference
11–20	6 (46%)	7 (54%)	**0.027**
21–30	2 (29%)	5 (71%)	0.212
> 30	1 (17%)	5 (83%)	0.554

COPD = chronic obstructive pulmonary disease, SD = standard deviation, VTE = venous thromboembolism

Positive outcome: treatment on regular ward, or discharge

Negative outcome: treatment on ICU, mechanical ventilation, ECMO, or death

* occasional missing values

** for 11 patients symptom onset was not documented

Patients with VTE were statistically significant more likely to experience a negative clinical outcome than patients without evidence of VTE (Table 4). Moreover, scans performed 11–20 days after symptom onset revealed a significant higher number of VTE than those scans were performed 0–10 days after symptom onset (p = 0.027).

#### CT findings

**Fig 2:**
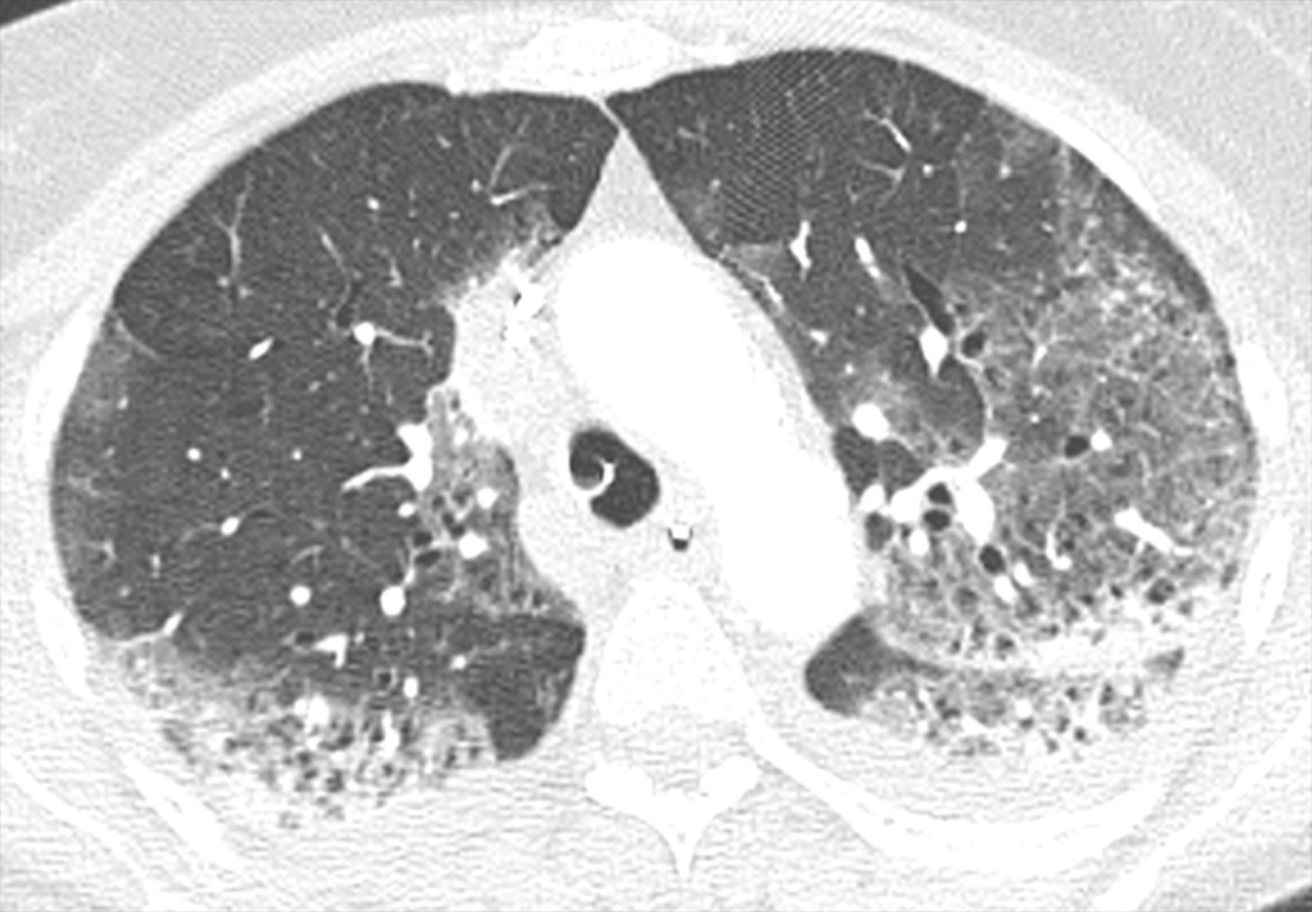
Crazy paving. Axial reconstruction of a CT in lung window demonstrating crazy paving.

**Fig 3:**
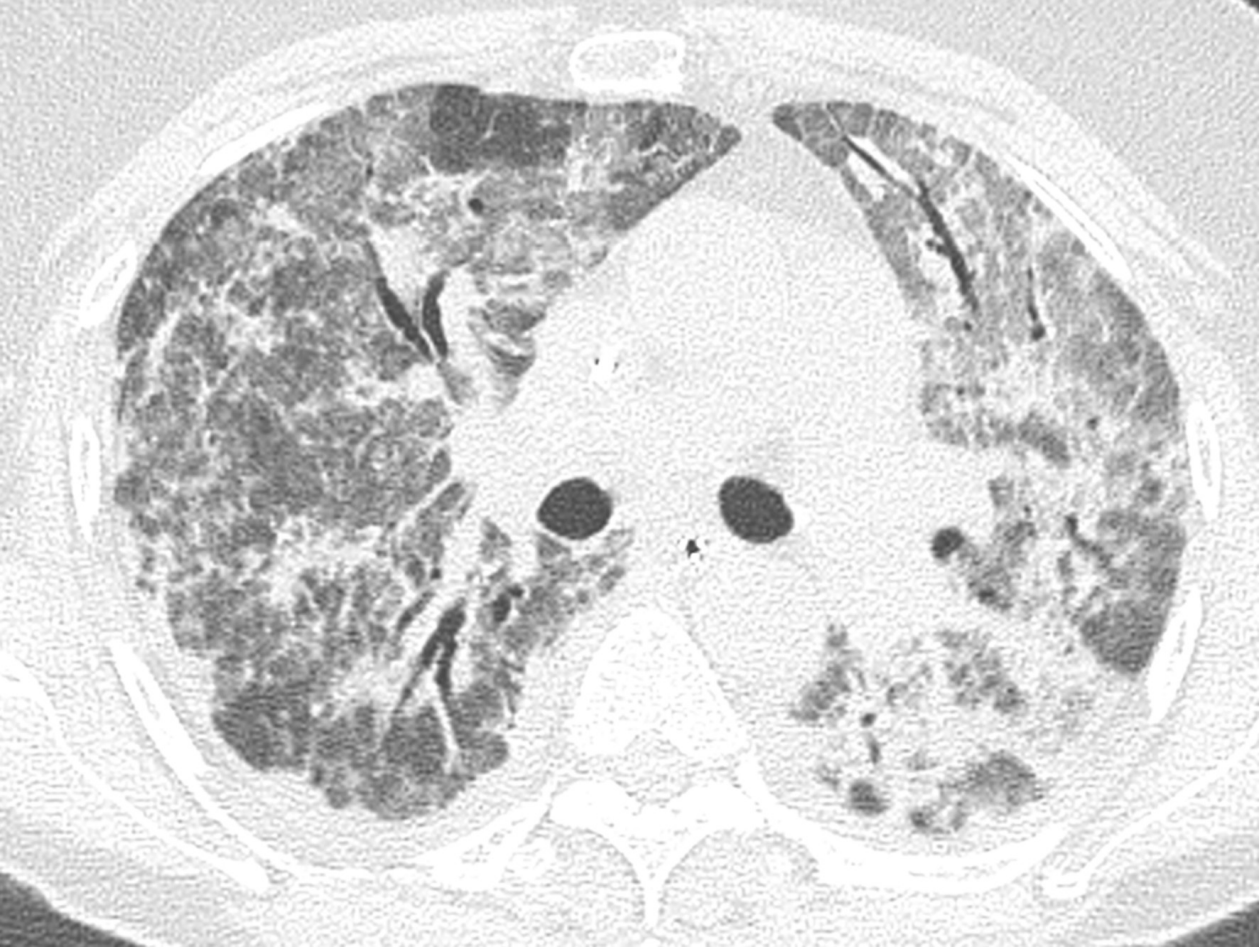
Air bronchogram. Axial reconstructions of a CT in lung window showing air bronchogram.

Crazy paving pattern (p = 0.024) (Fig 2) and air bronchogram (p = 0.021) (Fig 3) were correlated with incidence of VTE. Apart from these two findings, no other CT findings were associated with VTE (Table 5).

**Table 5:** Differences between VTE vs. no VTE regarding CT features.

CT findings	VTE group (n = 14)	no VTE group (n = 36)	p-value
GGO			
no	1 (50%)	1 (50%)	
yes	13 (27%)	35 (73%)	0.479
consolidation			
no	1 (14%	6 (86%)	
yes	13 (30%)	30 (70%)	0.384
crazy paving			
no	4 (15%)	23 (85%)	
yes	10 (43%)	13 (57%)	**0.024**
round shape of opacification			
no	7 (22%)	25 (78%)	
yes	7 (39%)	11 (61%)	0.198
sharp margin of opacification			
no	1 (8%)	12 (92%)	
yes	13 (35%)	24 (65%)	0.058
geographic shape of opacification			
no	9 (24%)	29 (76%)	
yes	5 (42%)	7 (58%)	0.226
curvilinear/bandlike opacification			
no	10 (31%)	22 (69%)	
yes	4 (22%)	14 (78%)	0.495
bronchial dilatation			
no	10 (29%)	25 (71%)	
yes	4 (27%)	11 (73%)	0.891
air bronchogram			
no	2 (10%)	18 (90%)	
yes	12 (40%)	18 (60%)	**0.021**
cavitation			
no	0 (100%)	0 (100%)	*---*
vessel enlargement			
no	4 (18%)	18 (82%)	
yes	10 (36%)	18 (64%)	0.171
pleural effusion			
no	9 (26%)	26 (74%)	
yes	5 (33%)	10 (67%)	0.582
lymphadenopathy			
no	8 (38%)	13 (62%)	
yes	6 (21%)	23 (79%)	0.176
unilateral			
no	14 (29%)	35 (71%)	
yes	0 (0%)	1 (100%)	0.529
bilateral			
no	1 (33%)	2 (67%)	
yes	13 (28%)	34 (72%)	0.832
right upper lobe			
no	1 (20%)	4 (80%)	
yes	13 (29%)	32 (71%)	0.675
right middle lobe			
no	1 (20%)	4 (80%)	
yes	13 (29%)	32 (71%)	0.675
right lower lobe			
no	1 (33%)	2 (67%)	
yes	13 (28%)	34 (72%)	0.832
left upper lobe			
no	1 (33%)	2 (67%)	
yes	13 (28%)	34 (72%)	0.832
left lower lobe			
no	1 (33%)	2 (67%)	
yes	13 (28%)	34 (72%)	0.832
peripheral			
no	8 (33%)	16 (67%)	
yes	6 (23%)	20 (77%)	0.420
central			
no	14 (28%)	36 (72%)	*---*
diffuse			
no	7 (27%)	19 (73%)	
yes	7 (29%)	17 (71%)	0.860
predominantly anterior			
no	14 (28%)	36 (72%)	---
predominantly posterior			
no	2 (18%)	9 (82%)	
yes	12 (31%)	27 (69%)	0.412
extent of lung involvement			
mild	1 (12%)	7 (88%)	reference
moderate	0 (0%)	6 (100%)	*0*.*369*
severe	12 (35%)	22 (65%)	0.210

CT = computed tomography, GGO = ground-glass opacities

#### Laboratory parameters

NT-pro BNP (p = 0.043), CK (p = 0.023) and D-dimers (p = 0.035) were correlated with VTE. There was also a tendency towards an elevated lymphocyte count in the group of VTE, which, however, did not reach statistical significance (p = 0.057) (Table 6).

**Table 6:** Differences between VTE vs. no VTE regarding laboratory parameters.

parameter	VTE group (n = 14)	no VTE group (n = 36)	p-value
CRP (mg/L)	152 (IQR 73.38–264.25)	112 (IQR 50.6–228.25)	0.292
leukocytes (/nl)	11.18 (IQR 8.34–17.88)	8.44 (IQR 5.82–12.71)	0.057
lymphocytes rel (%)	8.8 (IQR 5.9–16)	13 (IQR 8.3–21.4)	0.259
lymphocytes abs	1.08 (IQR 0.76–1.47)	1.09 (IQR 0.81–1.53)	0.987
eosinophiles rel (%)	0.2 (IQR 0–0.8)	0.4 (IQR 0–1.55)	0.506
eosinophiles abs	0.04 (IQR 0.01–0.09)	0.06 (IQR 0–0.19)	0.737
troponine (ng/l)	68.4 (IQR 18.2–115)	18.1 (IQR 13.6–38.6)	0.132
NT-proBNP (pg/ml)	2103 (IQR 1682–5293)	508 (IQR 387–923)	**0.043**
fibrinogen (mg/dl)	532.7 (IQR 397.25–631.45)	602 (IQR 536.3–688.3)	0.171
interleukin 6 (pg/ml)	122.7 (IQR 60.1–558.1)	157.9 (IQR 49.85–439.2)	0.803
D-dimer (mg/L)	8.75 (IQR 2.49–13.39)	2.35 (IQR 1.16–4.24)	**0.035**
LDH (U/I)	415.5 (IQR 293.5–540.5)	392 (IQR 306–458)	0.554
CK (U/I)	392.5 (IQR 168.5–1136)	97 (IQR 54.5–255.75)	**0.023**
Ckmb (ng/ml)	3.1 (IQR 1.88–9.8)	2.75 (IQR 1.75–6.53)	0.812
lactate (mg/dl)	10 (IQR 9–15)	12 (IQR 9–13.75)	0.834

CRP = C-reactive protein, NT-proBNP = N-terminal pro-B-type natriuretic peptide, LDH = lactate dehydrogenase, CK = creatin-kinase, CKmb = creatin-kinase muscle brain

## Discussion

As the lung is the main target organ of SARS-CoV-2, chest CT plays a major role in the care of patients with COVID-19. According to international guidelines for imaging in COVID-19, the standard care is low-dose chest CT without administration of contrast agent [11, 12]. Thus, pulmonary embolism might be undetected at CT. Meanwhile we had to learn that COVID-19 is more frequently complicated by VTE than other pneumonias [7–10, 13, 14]. Several studies assessed the predictive significance of CT in COVID-19 with regard to patient outcome in general [15–20], but none particularly examined CT characteristics of COVID-19 pneumonia in the light of VTE.

In our cohort of 50 patients with RT-PCR proven COVID-19 the percentage of patients with VTE detected by CT was 28% and thus is in the range of previously reported data [6–10, 13, 21]. Of note, the chest CTs performed on day 11–20 after symptom onset revealed a significant higher number of PE (46%) compared to CTs done on day 0–10. This suggests that patients in this stage of disease might bear a higher risk for VTE as compared to patients examined earlier or later in the disease course. Obviously the development of hypercoagulability takes some time as is supported by literature data showing rising D-Dimer levels during the course of disease [22].

Remarkably, only 2 out of 14 patients (14.3%) affected by PE had evidence of DVT, and only 4 out of the 14 patients (28.6%) with PE were examined for DVT based on clinical suspicion. This suggests that most of VTE events were unexpected PE and not accompanied by clinical signs of DVT. Our finding is in accordance with another publication [23] and supports the hypothesis of local (“in situ”) thrombus formation being a characteristic of COVID-19 associated disturbance of the coagulatory system rather than classical venous thromboembolism due to remote thrombogenesis [24].

Comparing comorbidities and clinical symptoms assessed at initial presentation of patients without and with VTE, we could not identify any clinically relevant significant differences. Thus, there seem to be no anamnestic or clinical findings which could serve as a red flag indicating an increased risk for VTE in COVID-19.

Laboratory parameters indicating inflammation (C-reactive protein, interleukin-6 and leucocyte count) were similarly elevated in the VTE and non-VTE group, thus the extent of inflammatory activity does not seem to correlate with the risk for VTE. Expectably, D-dimer values of patients with VTE were significantly higher than those of patients without evidence of VTE (p = 0.035), and NTproBNP and CK values were significantly elevated in the VTE group (p = 0.043 and 0.023, respectively), indicating right heart strain.

In our study, crazy paving pattern and air bronchogram on chest CT were significantly more frequent in patients with VTE than in patients without evidence for VTE (p = 0.024 and 0.021, respectively). 92% of patients with VTE had a severe extent of pulmonary involvement as compared to 63% of patients without evidence of VTE. Although the difference was not statistically significant, CT in patients with VTE in most cases reflected a more advanced stage of pulmonary involvement in COVID-19. We think that this finding might explain the higher incidence of a crazy paving pattern in CT of patients with VTE, as crazy paving usually is visible in more severe and advanced lung disease [25–27]. This might also apply to the higher prevalence of air bronchogram in CT of patients with VTE. Except these two findings we could not identify any other imaging characteristics that appeared more frequently in the context of VTE other than the direct proof of filling defects in the pulmonary vasculature. Remarkably, there was no preponderance of peripheral or posterior lung involvement (which are hallmarks of pulmonary infarction) in the VTE group as compared to the non-VTE group. As the incidence of vessel enlargement did not differ in both groups (VTE vs. non-VTE), we do not assume that this phenomenon is related to PE. Regarding our CT findings of COVID-19 pneumonia complicated by VTE, both crazy paving pattern and / or air bronchogram on a non-contrast-enhanced chest CT could give a hint on occult PE.

In our study, the vast majority (93%) of patients with VTE had a negative clinical outcome (i.e. need for treatment on ICU, mechanical ventilation, ECMO, or death), whereas without evidence of VTE, 61% of patients had a negative outcome. This difference was statistically significant (p = 0.028).

Our study has limitations. CT scans were performed at the discretion of the referring physician. Because we focused on pulmonary embolism, we only could include patients who underwent contrast-enhanced CT. Hence, a bias towards seriously ill patients has to be assumed, since contrast-medium application usually is reserved for this group of patients with COVID-19. The recruiting hospitals were tertiary care centers with one of them being the regional main center for critically ill patients. Thus, the proportion of patients with severely affected lung parenchyma was comparatively high. Further research is mandatory to evaluate long term prognosis and possible residua after restitution.

## Conclusion

Patients with COVID-19 pneumonia who underwent contrast-enhanced CT had signs of PE in a substantial percentage (28%) and, remarkably, did not have evidence of concomitant DVT in the vast majority of thus affected patients. As we found the incidence of PE to be significantly higher when CT was performed on day 11–20 after symptom onset, we call for a high level of suspicion for VTE particularly in this time period and advocate a lower threshold for contrast-agent administration at chest CT in COVID-19 pneumonia. According to our observations this might especially be justified for follow-up examinations if previous non-enhanced CT shows a crazy-paving pattern and / or air bronchogram, as these two imaging characteristics were significantly associated with PE in our study.

## Supporting information

S1 File (XLSX)
